# Advertisers, &c.

**Published:** 1889-03

**Authors:** 


GLYCERINE SUPPOSITORIES.—The glycerine suppositories recently brought out by Messrs. Parke, Davis & Co. must prove a very convenient and acceptable device for the practitioner. In a note from the proprietors accompanying a sample of these suppositories they remark:

"For a long while glycerine has been successfully used in Europe as an enema, to provoke alvine evacuations, but owing to its physical and chemical properties difficulties have been heretofore encountered in presentiug it in an acceptable form which would dispense with the injection apparatus.

Under these circumstances we began a series of experimentations to produce glycerine more eligibly for enemas, and as the result of our labors we now present to the medical profession "Glycerine Suppositories." Thinking you might like to test them, we send you specimens, by mail, to-day, with our compliments. We believe Doctor, that you will find these suppositories to be useful, especially in stubborn cases where the contents of the lower bowel are hard to move by the ordinary methods.

We will be unable to present samples of the suppositories to the general profession, so we would ask that you will kindly speak of them to your medical friends at some opportune moment, when should they express a wish for specimens, if they will address us we shall be glad to transmit them.

LIQUOR CARBONIS DETERGENS is a solution, or rather an emulsion of coal tar which is miscible with water. It can generally be obtained of the druggists, and is made in this country by L. Wolff, of Philadelphia, and probably some other manufacturers. Useful in skin affections and a cleansing wash for ulcers.

JACOBS' PHARMACY CO.—This is a wholesale and retail drug House on the corner of Marietta and Peachtree Sts., Atlanta. The proprietors are active, accommodating and energetic business men. They have established a reputation for low prices and prompt attention to orders sent them. See their advertisement in this Journal.

### BOOKS AND PAMPHLETS RECEIVED.

HAND BOOK of Materia medica, pharmacy and therapeutics, compiled for the use of students preparing for examination by Cuthbert Bowen, M. D., B. A. Editor of "Notes on practice," Philadelphia and London. F. A. Davis, Publisher. 1888.

This is a very condensed and valuable resume of the drugs recognized by the United States Pharmacopœia, and all the officinal and important preparations. It is in the form of qnestion and answer, and is well adapted to the wants of the student who is preparing for the green room. It contains much valuable information as to methods of study, prescription writing, abbreviations, weights and measures, incompatibilities, table of drops, definitions, methods of administration, dosage, antiseptics etc. etc. 366 octavo pages, neatly printed.

INTERNATIONAL POCKET MEDICAL FORMULARY, with an appendix containing posological table. Formulæ for inhalations, suppositories, nasal douches, eye washes and gargles; hypodermic formulæ, table of hypodermic medication, use of thermometers in disease; poisons and their antidotes, post mortem and medico legal examinations, artificial respiration, ligation of arteries; obstetrical table, urinalysis, differential diagnosis of eruptive, typhoid aud typhus fevers, tables of pulse; temperature; respiration, motor points, etc., by Sumner Witherstine, M. S., M. D., associate editor, Annual of Universal Medical Sciences, late House surgeon Charity Hospital, New York, etc., Philadelphia and London. F. A. Davis, publisher. 269 pages—Price $2.00, net.

This is a neatly bound and elegantly gotten up pocket formulary and useful memoranda, etc., with select formulæ, marginal index and subjects alphabetically arranged, very practical and convenient for the practitioner.

OSTEOTOMY for anterior curves of the leg, by De Forest Willard, M. D., Lecturer on Orthopædic surgery, University of Pennsylvania, etc. Read before the American Orthopædic Society, at Washington, September, 1888.

